# Dermatomyositis with positive anti‐TIF1 gamma antibodies in an adult female: A case report

**DOI:** 10.1002/ccr3.8215

**Published:** 2023-11-20

**Authors:** Krishna Adhikari, Prashant Pant, Sanjeev Bhandari, Sandip Paudel, Buddhi Poudyal, Lucky Sharma, Keshav Raj Sigdel, Rojina Subedi

**Affiliations:** ^1^ Patan Academy of Health Sciences Lalitpur Nepal; ^2^ Department of Internal Medicine Star Hospital Lalitpur Nepal; ^3^ Department of Internal Medicine Karnali Province Hospital Birendranagar Nepal; ^4^ College of Medicine Nepalese Army Institute of Health Sciences Kathmandu Nepal; ^5^ Pokhara Academy of Health Sciences Pokhara

**Keywords:** autoimmune, dermatomyositis, malignancy, Nepal, TIF‐1

## Abstract

Dermatomyositis is an uncommon autoimmune disease with only few cases reported from Nepal. Presence of anti TIF‐1 gamma antibodies in DM are the strongest predictor of malignancy. Timely screening of malignancies for early detection and management remains the mainstay of this report.

## Graphical abstract

Clinical features suggestive of dermatomyositis.

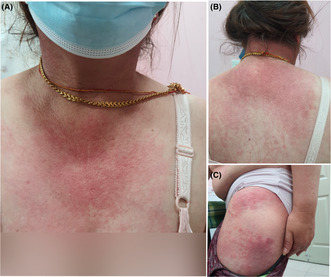

## 1 INTRODUCTION

Idiopathic inflammatory myopathy (IIM), is an umbrella term that includes a group of autoimmune disorders with varying clinical manifestations. Dermatomyositis (DM) lies alongside antisynthetase syndrome, inclusion body myositis, immune mediated necrotizing myopathy, overlap myositis and is an uncommon IIM.^1^, ^2^

The estimated prevalence of DM is 1 per 100,0000 populations.^3^ It typically peaks at two age groups, the juvenile DM at 5–18 years and adult DM at 45–64 years of age. Females are almost affected twice compared to males.^4^Anti TIF‐1 gamma antibodies are found in 7%–41% of patients with DM.^5^The relationship between these antibodies and malignancy have been well established and prevalence of malignancy ranges from 38% to 71%. Thus, Anti TIF‐1 gamma antibodies remain the strongest predictor of malignancy in individuals with DM.^5^, ^6^

Very few cases of DM have been reported from Nepal and none with positive anti TIF‐1 gamma antibodies to the best of our knowledge. We present a case of a 54‐year‐old female with dermatomyositis having positive anti TIF‐1 gamma antibodies. Screening at appropriate intervals and timely diagnosis of possible malignancies in individuals with anti TIF 1 gamma antibodies remains the mainstay of this report.

This report is in line with CARE guidelines.^7^

## 2 CASE REPORT

A 50‐year‐old female without any comorbidities in the past, presented to OPD with complaints of pruritic rashes involving face, dorsum of fingers, upper extremities, upper back and front of torso, scalp, and lateral aspects of thigh for 2 months. The lesions get worsened with sunlight which has led to frequent abstinence from her workplace. In addition, she also experienced muscle weakness for 20 days. Concurrently she has easy fatigability and feverish feeling. She is the first in her family to experience such symptoms.

### 2.1 Diagnostic assessments

**FIGURE 1:**
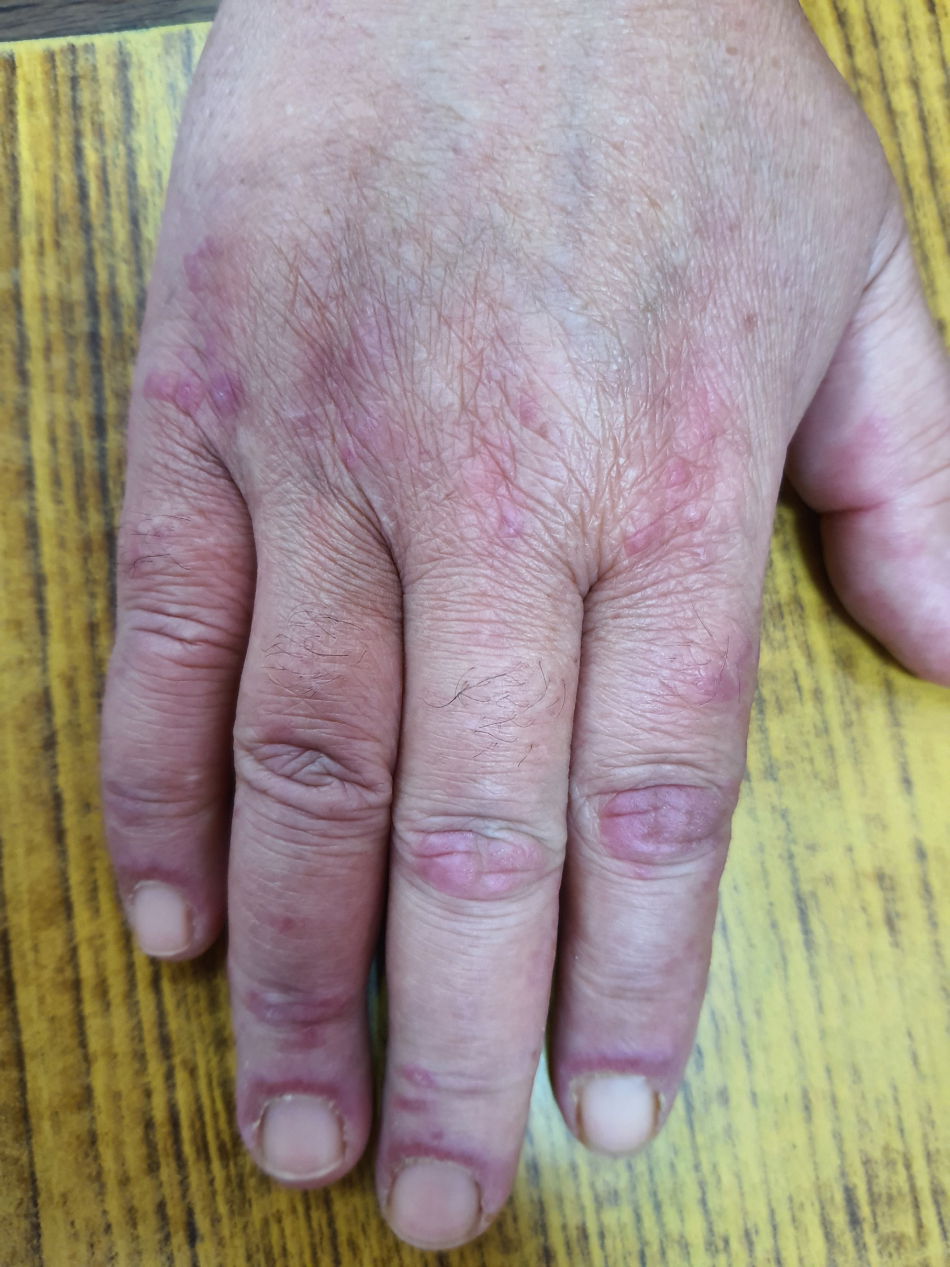
Figure showing Gottron's papules pathognomic of dermatomyositis.

**FIGURE 2:**
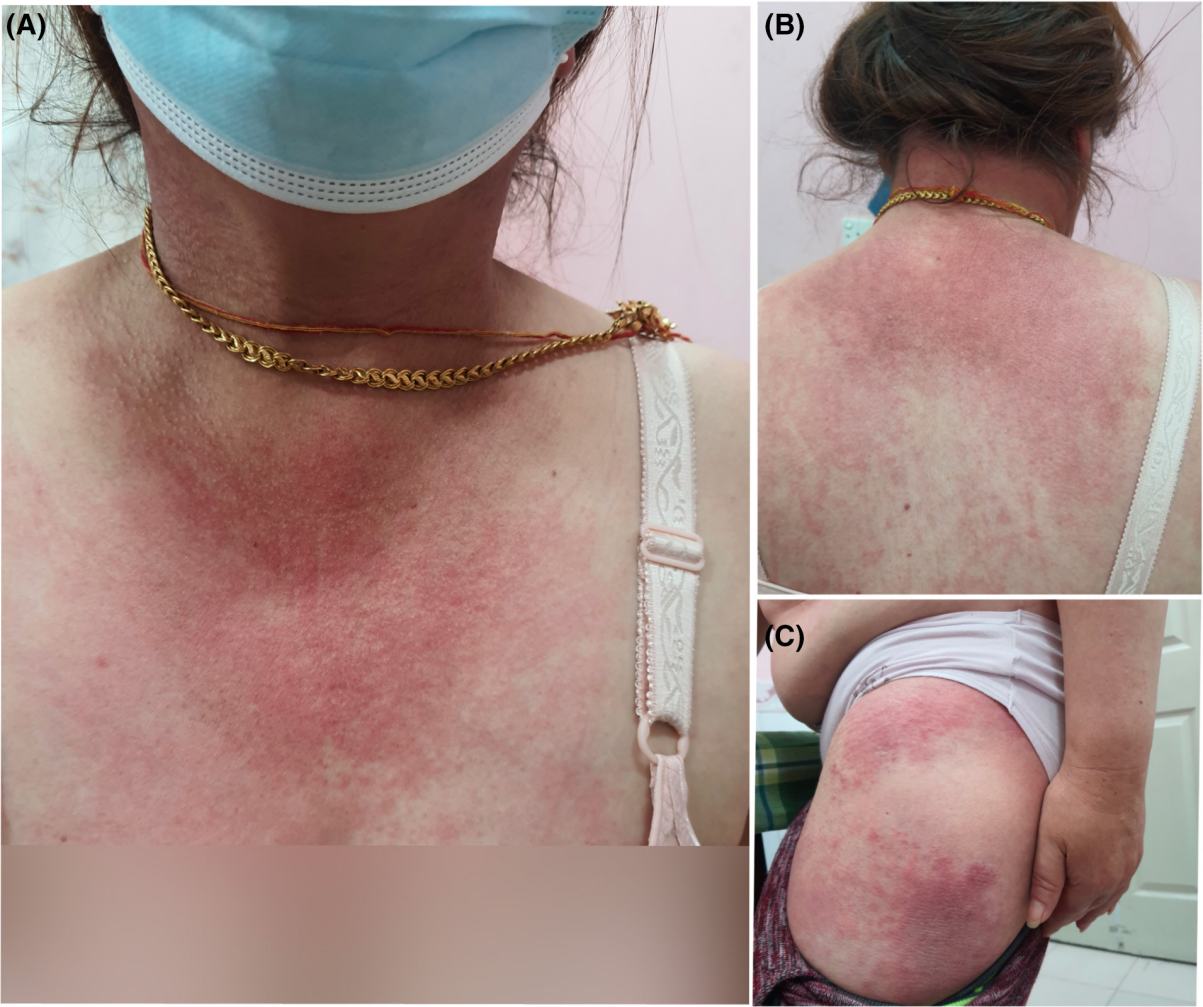
(A) Figures showing V sign, (B) Shawl sign and (C) Holster sign respectively.

On examination, the patient was conscious, well oriented with stable vitals. Mucocutaneous examination revealed Violaceous erythematous rashes present on the periorbital skin, forehead, chin, cheek malar prominence without sparing nasolabial fold. Gottron's papules, which are multiple violaceous, non‐scaly papules present over the joints on the dorsum of hand were seen. (Figure 1) Additionally, V sign (Figure 2A), Shawl sign (Figure 2B), Holster sign (Figure 2C), and gottron's sign were evident on examination. Moreover, the patient had proximal muscle weakness when muscle strength was evaluated. She had 4/5 power in flexor and extensor of neck; all groups of shoulder and hip muscles. However, there was no evidence of any respiratory muscle weakness.

On grounds of suspicion of rheumatological disorder based on clinical findings, investigations were sent including complete and differential blood counts, liver function test, Renal function test, muscle enzymes, and antibody tests with myositis panel antibody tests. Elevation in CK levels and strong positive anti TIF‐1 gamma antibodies were found. The findings of investigations are summarized in the table below (Table 1). A final diagnosis of dermatomyositis was made on the basis of history, clinical examination, and supportive investigation.

**TABLE 1:** Summary of investigation findings.

Parameter	Values	Parameter	Values
Total count	7100	**CK total**	226
Differential count	N74, L23	Rheumatoid factor (RF)	negative, Anti‐CCP: 4.12 (<20: negative)
**Hb/Hct**	**11/34**	ANA by ELISA	0.5 (<1 Normal)
ESR/CRP	14/5	ANA by IFA	1:80, Homogenous nuclear pattern
Urea/Creatinine	12/0.8	**Myositis panel antibodies:**	**TIF 1 gamma‐ strong positive**
Urine R/E	Normal	Autoantibody Panel	All negative
SGPT/SGOT	45/36	Pap Smear	Negative for intraepithelial lesion or malignancy

*Note*: Highlightings in the text denotes raised CK levels and Strong positive anti TIF‐1 gamma antibodies.

### 2.2 Management

The patient was started on oral prednisolone 1 mg/kg/day for 4 weeks followed by plan of tapering to 10 mg/day at the end of 12 months. Additionally, methotrexate was started at 7.5 mg/week and titrated to 20 mg/week within 6 weeks. Concurrently, supportive therapy with calcium, vitamin D and co‐trimoxazole 480 mg were given. In view of positive TIF 1 gamma, malignancy screening was persuaded which were negative. However, we will be screening for malignancy every alternate year.

### 2.3 Follow‐up

The patient is under regular follow up and her symptoms are improving. Screening for malignancy done during the follow up visit showed no evidence of malignancy.

## 3 DISCUSSION

Dermatomyositis is an uncommon autoimmune disease with different clinical phenotypes. Skin and muscle involvement are the major clinical entities and is more frequently found in Caucasians compared to the Asians.^8^

Genetic association studies have revealed the linkage of MHC genetic mutations todermatomyositis. That being said, DM possesses additional non MHC genetic risks too.^9^ The progress in genetic mapping of DM has been hampered due to it rare occurrence. In genetically susceptible individuals, environmental factors like UV exposure, drugs and lifestyle choices may precipitate the condition.^10^ Qiang et al. found in their study that the risk of malignancy among individuals with dermatomyositis is increased by 4.66‐folds compared to the general population.^5^ Patients with anti TIF‐1 gamma antibodies have strongest association with cancer with 9.37‐fold higher risk.^11^ This necessitates the importance of cancer screening in individuals testing positive for anti TIF‐1 gamma antibodies. Our patient is planned for biannual screening for possible malignancies including ovarian, lungs, stomach and colorectal cancers.

Clinically, patients present with typical cutaneous manifestations like Gottron's papules and Heliotrope rashes. Gottron's papules are violaceus non erythematous papules present over metacarpophalangeal joints and interphalangeal joints whereas heliotrope rashes are present on periorbital skin, forehead, chin etc.^12^ Our patient had typical Gottron's papules and Heliotrope rashes and additional features like erythema of posterior neck (Shawl sign), knee and elbow (gottrons's sign), anterior upper chest(V sign), Holster sign. Additionally, features of muscle weakness were present in our patient as she complained of difficulty in climbing stairs, doing daily chores and even transforming herself from sitting to standing position. Besides cutaneous and muscle manifestations, involvement of lungs, joints, heart etc. may be found but our patient denied such symptoms.

The 2017 EULAR criteria is the current standard for diagnosis of IIM. This criterion includes a classification tree that identifies and sub‐classifies IIM. The sub‐classes includepolymyositis; immune‐mediated necrotizing myopathy; inclusion body myositis; amyopathicdermatomyositis; dermatomyositis, and juvenile dermatomyositis. This classification criterion has the highest sensitivity and specificity in diagnosing IIM.^13^Based on this classification criteria, our case falls under definite IIM, and sub‐class dermatomyositis. The clinical features and classification criteria helped in the diagnosis of the case and treatment was initiated accordingly.

Due to lack of validated clinical outcomes from randomized control trials, the food and drug administration has not approved any standard treatment for dermatomyositis. Initial therapy includes aggressive photoprotection, antipruritic agents and topical therapy with corticosteroids.^14^, ^15^ Glucocorticoids remain the mainstay for treatment of DM. Disease Modifying Anti Rheumatic Drugs (DMARDs), intravenous immunoglobins, other biologics and topical antibiotics are other treatment options.^14^ In our case, oral prednisolone 1 mg/kg/day was started for 4 weeks followed by plan of tapering to 10 mg/day at the end of 12 months. Additionally, methotrexate was started at 7.5 mg/week and titrated to 20 mg/week within 6 weeks. Concurrently, supportive therapy with calcium, vitamin D and co‐trimoxazole 480 mg was initiated.

In addition to pharmacological therapy, screening for malignancy remains an important aspect in management of anti TIF‐1 gamma positive dermatomyositis. In order to maximize the benefits of screening, the individuals should be classified as high‐risk subgroup for developing malignancy.^16^ In view of positive anti TIF 1 gamma, the strongest predictor of malignancy in dermatomyositis patients, screening was persuaded which were negative. We will be screening for malignancy biannualy in the patient in subsequent follow‐up visits.

## 4 CONCLUSION

Pharmacological management of dermatomyosits is a priority but screening at appropriate intervals and timely diagnosis of possible malignancies in individuals with anti TIF 1 gamma antibodies remains the mainstay of this report.

### 4.1 Patient's perspective

The patient was anxious about her condition as she had frequent abstinence from her workplace owing to the disease. After symptomatic relief, she is satisfied and has returned to her regular workplace.

## AUTHOR CONTRIBUTIONS

**Krishna Adhikari:** Conceptualization; data curation; formal analysis; investigation; methodology; project administration; resources; supervision; visualization; writing – original draft; writing – review and editing. **Prashant Pant:** Conceptualization; data curation; formal analysis; project administration; supervision; visualization; writing – original draft; writing – review and editing. **Sanjeev Bhandari:** Conceptualization; formal analysis; methodology; project administration; supervision; validation; writing – review and editing. **Sandip Paudel:** Conceptualization; data curation; project administration; resources; visualization; writing – original draft; writing – review and editing. **Buddhi Poudyal:** Data curation; investigation; methodology; project administration; resources; supervision. **Lucky Sharma:** Conceptualization; methodology; resources; supervision; writing – review and editing. **Keshav Raj Sigdel:** Conceptualization; formal analysis; resources; writing – review and editing. **Rojina Subedi:** Data curation; validation; writing – review and editing.

## FUNDING INFORMATION

This article did not receive any grants.

## CONFLICT OF INTEREST STATEMENT

There is no conflict of interest.

## ETHICS STATEMENT

Not applicable.

## CONSENT

Written informed consent was obtained from the patient for publication of this case report and accompanying images. A copy of the written consent is available for review by the Editor‐ in‐ Chief of this journal on request.

## Data Availability

The data used to support the findings of this study are included within the article.
